# Visualization of autoantibodies and neutrophils *in vivo* identifies novel checkpoints in autoantibody-induced tissue injury

**DOI:** 10.1038/s41598-020-60233-w

**Published:** 2020-03-11

**Authors:** Jennifer E. Hundt, Hiroaki Iwata, Mario Pieper, Rebecca Pfündl, Katja Bieber, Detlef Zillikens, Peter König, Ralf J. Ludwig

**Affiliations:** 1 Institute of Experimental Dermatology, University of Lübeck, Lübeck, Germany; 2 Department of Dermatology, University of Lübeck, Lübeck, Germany; 3 Insitute of Anatomy, University of Lübeck, Germany and Airway Research Center North (ARCN), Member of the German Center for Lung Research (DZL), Lübeck, Germany; 4 Present Address: Department of Dermatology, Hokkaido University Graduate School of Medicine, Sapporo, Japan

**Keywords:** Autoimmunity, Experimental models of disease

## Abstract

In several autoimmune diseases, e.g., pemphigoid disease (PD), autoantibodies are the direct cause of pathology. Albeit key requirements for antibody-mediated diseases were identified, their interactions and exact temporal and spatial interactions remained elusive. The skin is easily accessible for imaging. Thus, we selected epidermolysis bullosa acquisita (EBA), a PD with autoantibodies to type VII collagen (COL7), to visualize interactions of autoantibodies, target tissue and effector cells (neutrophils). Following injection into mice, anti-COL7 IgG bound to the dermal-epidermal junction (DEJ) within minutes. We unexpectedly observed an inhomogeneous distribution of autoantibodies along the DEJ. Thus, we hypothesized that specific external triggers may affect autoantibody distribution. Indeed, mechanical irritation led to an increased autoantibody binding along the DEJ. Subsequently, anti-COL7 IgG was injected into mice expressing green fluorescent protein under the LysM promoter (LysM-eGFP) mice. This allows to visualize myeloid cells *in vivo* in these animals. Using multiphoton imaging, we observed a limited extravasation of LysM-eGFP^+^ cells into skin was observed within 24 hours. Intriguingly, LysM-eGFP^+^ cells did not immediately co-localize with autoantibodies, which was only noted at later time points. Of note, interactions of LysM-eGFP^+^ with the autoantibodies at the DEJ were short-lived. Collectively, our results define the following checkpoints for autoantibody-induced tissue injury: (i) autoantibody egress to target tissue influenced by mechanical trigger factors, (ii) neutrophil recruitment into the vicinity of autoantibody deposits and (iii) short-term neutrophil localization to these deposits, as well as (iv) delayed recruitment of neutrophils with subsequent autoantibody-induced inflammation.

## Introduction

Autoimmune diseases are characterized by aberrant, specific immune responses against self-antigens^1^. Most autoimmune diseases have characteristic autoantibodies, i.e., anti-nuclear autoantibodies in patients with systemic lupus erythematous (SLE) or autoantibodies against citrullinated proteins in rheumatoid arthritis (RA)^2–4^. Thus, detection of these autoantibodies has become essential for diagnosis^5,6^. Moreover, a direct link regarding autoantibody binding has been demonstrated for several, but not all, autoimmune diseases. Well-established examples for this causal relationship include autoantibodies against thyroglobulin in autoimmune thyroiditis^7–9^ and glycoproteins IIb and IIIa in immune thrombocytopenia (ITP)^10,11^. Autoimmune skin blistering dermatoses (AIBD) comprise a good illustration of a direct pathogenic contribution of autoantibodies. In AIBD, blistering is caused by autoantibodies directed against specific structural components of the skin. The pathogenic activity of autoantibodies from patients with AIBD has been demonstrated by (i) induction of blistering in mice by the transfer of autoantibodies from patients^12–14^, (ii) reproduction of several AIBD via the immunization of mice with autoantigen^15,16^, and (iii) correlation of autoantibody titers in patients^17,18^. These criteria fulfill the revised Witebsky’s postulates for the definition of autoimmune diseases^1^.

The molecular requirements that lead to autoantibody-induced tissue injury are relatively well characterized. Autoantibodies may induce pathology via (i) inhibition or activation of pathological signaling of specific receptors, as exemplified by myasthenia gravis, Graves’ disease or pemphigus, respectively^19–21^, (ii) phagocytosis in primary ITP^22^, or (iii) formation of immune-complexes, which lead to Fc gamma receptor (FcγR)-dependent activation of leukocytes and tissue destruction, as demonstrated in RA and pemphigoid diseases (PD)^2,23,24^. The molecular events that lead to tissue damage in RA and PD have been extensively investigated. In both diseases, complement activation, cytokines, and myeloid cells are unequivocally required for clinical disease manifestation, as demonstrated by a complete protection from RA/PD induction in mice in which the respective pathways/cells were experimentally blocked^25–28^.

Nevertheless, the precise temporal and spatial interactions of these key molecules and cells remain largely elusive, i.e., kinetics of antibody deposition and their interactions with the target antigen, and subsequent binding of effector myeloid cells is completely unknown. Insights from mouse models of RA have indicated a rapid and specific localization of glucose-6-phosphate isomerase (GPI)-specific antibodies to distal joints in the front and rear limbs within minutes of intravenous injection, which was demonstrated by positron emission tomography^29^. Inflammatory cells in experimental RA have been visualized using SPECT/micro-CT imaging labeled nanobodies directed against the macrophage mannose receptor^30^ and via injection of radiolabeled cells^31^. Of note, the differential expression of endothelial adhesion molecules^32^ and production of reactive oxygen species^33^ may also be determined by using *in vivo* imaging in experimental RA. To our knowledge, a direct and simultaneous observation of autoantibodies and effector leukocytes within the tissue targeted by the respective autoantibodies has not been described to date. Insights into this process would enable a better understanding of the early events in the pathogenesis of autoantibody-mediated diseases, such as RA and PD.

Due to the relatively good accessibility of skin for *in vivo* multiphoton microscopy, we selected the PD epidermolysis bullosa acquisita (EBA) to visualize the interactions of autoantibodies with both the target antigen and the effector cells. In EBA, the autoimmune response is directed against the main component of the anchoring fibrils in the skin, namely, type VII collagen (COL7)^34^, and Gr-1^+^ myeloid cells are indispensable for blister induction^35^. For visualization of events that lead to blister formation in EBA, we injected fully pathogenic, affinity-purified, fluorescently labeled anti-mouse COL7 antibodies into mice that expressed eGFP under the control of the endogenous lysozyme M promoter (LysM-eGFP mice) indicating a green fluorescence of neutrophils and monocytes^36^. This experimental design enabled investigation of autoantibody interactions with both the target antigen and effector cells using multiphoton microscopy.

With this technique, we addressed the following main questions: What are the kinetics of (i) autoantibody deposition and (ii) neutrophil recruitment into the skin? Furthermore, we aimed to visualize the migratory behavior of eGFP^+^ myeloid cells following their extravasation into the skin.

## Results

### Generation of fully pathogenic fluorescently labeled anti-COL7 IgG

**Figure 1:**
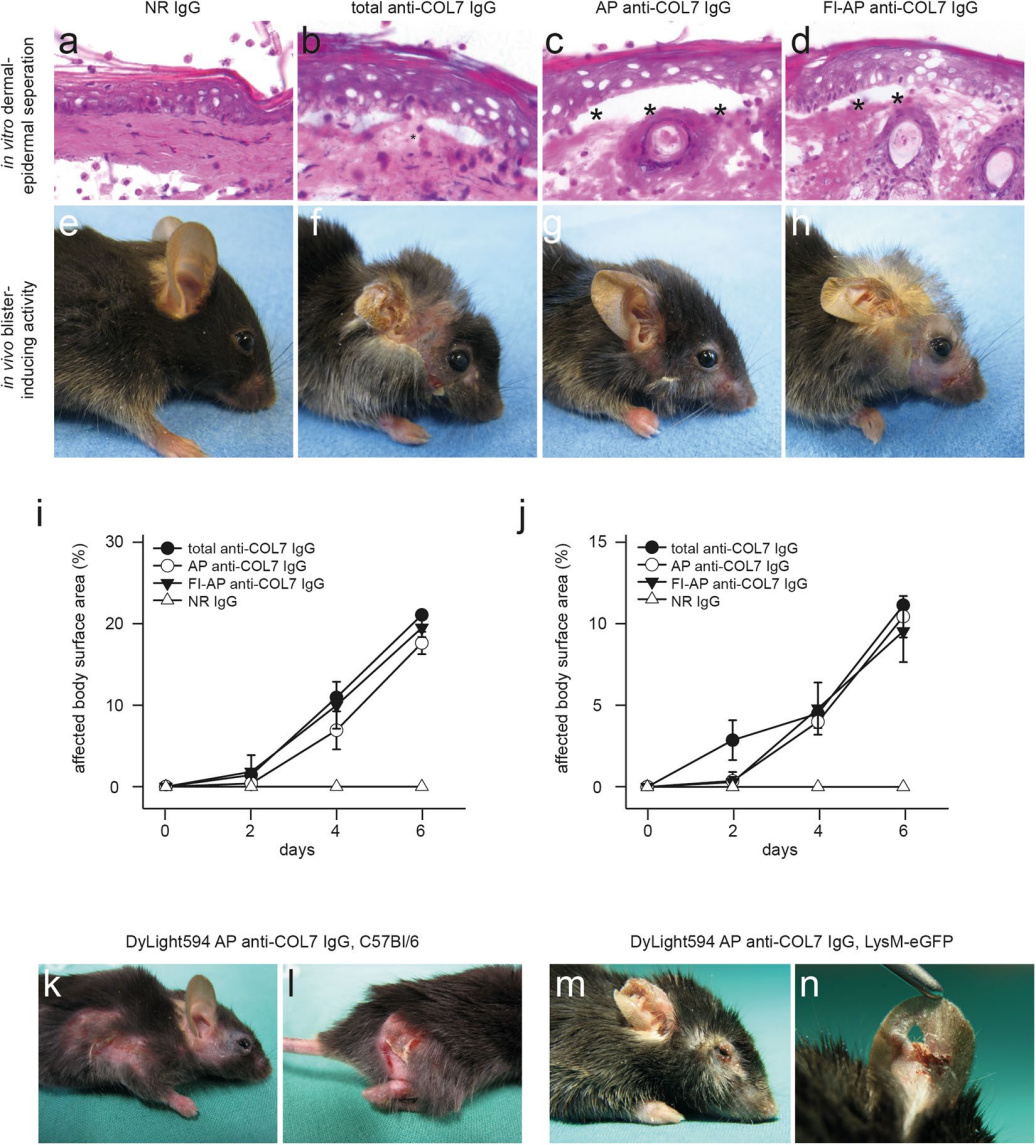
Pathogenicity of fluorescently-labeled, affinity-purified anti-COL7 IgG. (**a**–**d**) Sections of normal mouse skin were incubated with (**a**) normal rabbit IgG, (**b**) IgG purified form rabbits immunized with COL7, (**c**) affinity-purified (AP) anti-COL7 IgG, or (**d**) DyLight488 fluorescently-labeled (Fl), AP anti-COL7 IgG, followed by administration of peripheral polymorphnuclear cells. Similar concentrations of anti-COL7 IgG were added to conditions b-d, which induced *ex vivo* dermal-epidermal separation under all experimental conditions. (**e–h**) C57Bl/6 mice were s.c. injected with the indicated IgG preparations. Amount of anti-COL7 IgG was identical in conditions f-h, and induced a comparable extend of skin blistering, as demonstrated for immune preparations. (**i**) SA6307 and (**j**) SA6306. Data in i-j is based on 3-4 mice per group. (**k,l**) DyLight594-labelled AP anti-COL7 IgG was s.c. injected into a total of 3 C57Bl/6 mice. Representative clinical photographs of 2 of these mice obtained 12 days after the initial IgG injection are shown here, demonstrating extensive skin lesions. (**m**,**n**) DyLight594-labelled AP anti-COL7 IgG was s.c. injected into 3 LysM-eGFP mice. Representative clinical photographs of 2 of these mice obtained 12 days after the initial IgG injection are shown here. The data are expressed as the mean ± SEM. To compare the differences in the disease severity (AUC), independent samples Student’s t-tests were used. A p-value <0.05 was considered statistically significant.

Prior to use of anti-COL7 IgG preparations to visualize their interactions with the skin and neutrophils *in vivo*, they were assessed regarding their blister-inducing ability. Total IgG from GST-mCOL7C immune sera, affinity-purified (AP) IgG from these immune sera, and DyLight488- or DyLight594-labeled AP anti-COL7 IgG, but not normal rabbit IgG, exhibited linear depositions along the dermal-epidermal junction (DEJ) mouse skin. When incubated on cryosections of murine skin, all anti-COL7 IgG preparations induced dermal-epidermal separation in presence of polymorphonuclear leukocytes (PMN, Fig. 1a–d). If injected into C57Bl/6 mice, AP anti-COL7 IgG or DyLight488-labeled AP anti-COL7 IgG induced subepidermal blistering at a comparable degree (Fig. 1e–j) as previously reported for anti-COL7 IgG^37^. Similar findings were demonstrated when DyLight594-labeled AP anti-COL7 IgG was injected into C57Bl/6 or LysM-eGFP mice (Fig. 1k–n).

### Inhomogeneous distribution of anti-COL7 IgG along the dermal-epidermal junction

**Figure 2:**
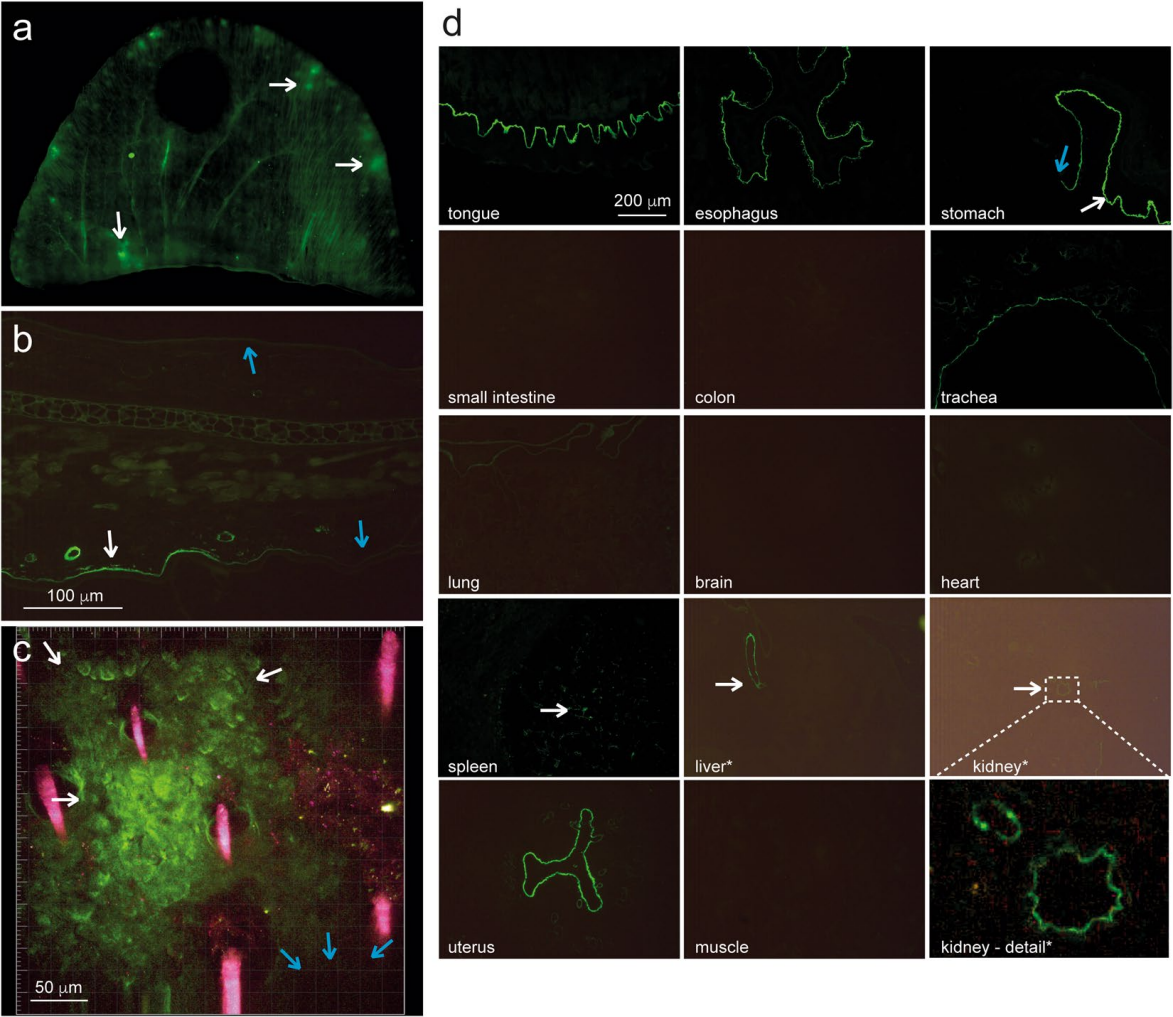
*In vivo* binding patterns of anti-COL7 IgG. (**a**) Fluorescent image of a total mouse ear obtained 1 hour after the i.v. injection of DyLight488-labeld anti-COL7 IgG. Anti-COL7 IgG is present in the vasculature (linear structures), as well as at extravascular localizations (white arrows). (**b**) Vertical section of an ear specimen obtained 10 hours after injection indicates anti-COL7 IgG deposition along the dermal-epidermal junction (DEJ) (white arrow). Interestingly, sites of the DEJ are completely devoid of autoantibody deposits (blue arrows). (**c**) These observations of an inhomogeneous and patchy binding of anti-COL7 IgG along the DEJ was confirmed by *in vivo* imaging via multiphoton microscopy. There are areas of anti-COL7 IgG deposits (white arrows) and areas devoid of IgG deposits (blue arrows); red structures indicate autofluorescent hair shafts. (**d**) Tissue distribution of a single injection of DyLight488-labeled, affinity purified anti-COL7 IgG in the indicated organs 10 hours thereafter. Experiments presented in a-d represent at least 3 experiments per condition.

An analysis of the DyLight488-labeled AP anti-COL7 IgG distribution following its intravenous injection indicated few extravascular deposits of IgG in the horizontal plane (Fig. 2a). In the vertical skin sections, the inhomogeneous anti-COL7 IgG distribution and binding to DEJ were confirmed (Fig. 2b). The observation of the anti-COL7 IgG deposition in real-time using multiphoton microscopy also reproduced this binding pattern. In addition, the real-time observation of anti-COL7 IgG following intravenous injection confirmed antibody binding to the DEJ within minutes (Fig. 2c). Collectively, these findings demonstrate a rapid but circumscribed binding of circulating anti-COL7 IgG to its target antigen.

We also investigated the binding pattern of DyLight488- or DyLight594-labeled AP anti-COL7 IgG 20 hours after a single injection into mice. In addition to the skin, linear IgG deposits were identified in the tongue and esophagus, as well as the epithelium of the stomach, trachea and uterus (Fig. 2d). A granular staining pattern was identified in the spleen, whereas circular structures were stained in the liver and kidney (Fig. 2d). No staining was identified in the small intestine, colon, lung, brain, heart or muscle (Fig. 2d). Control sections (injection of DyLight488- or DyLight594-labeled unspecific antibody) did not exhibit specific staining (supplementary Fig. S1).

### Mechanical irritation increases anti-COL7 IgG localization into the skin and affects cutaneous blistering

**Figure 3:**
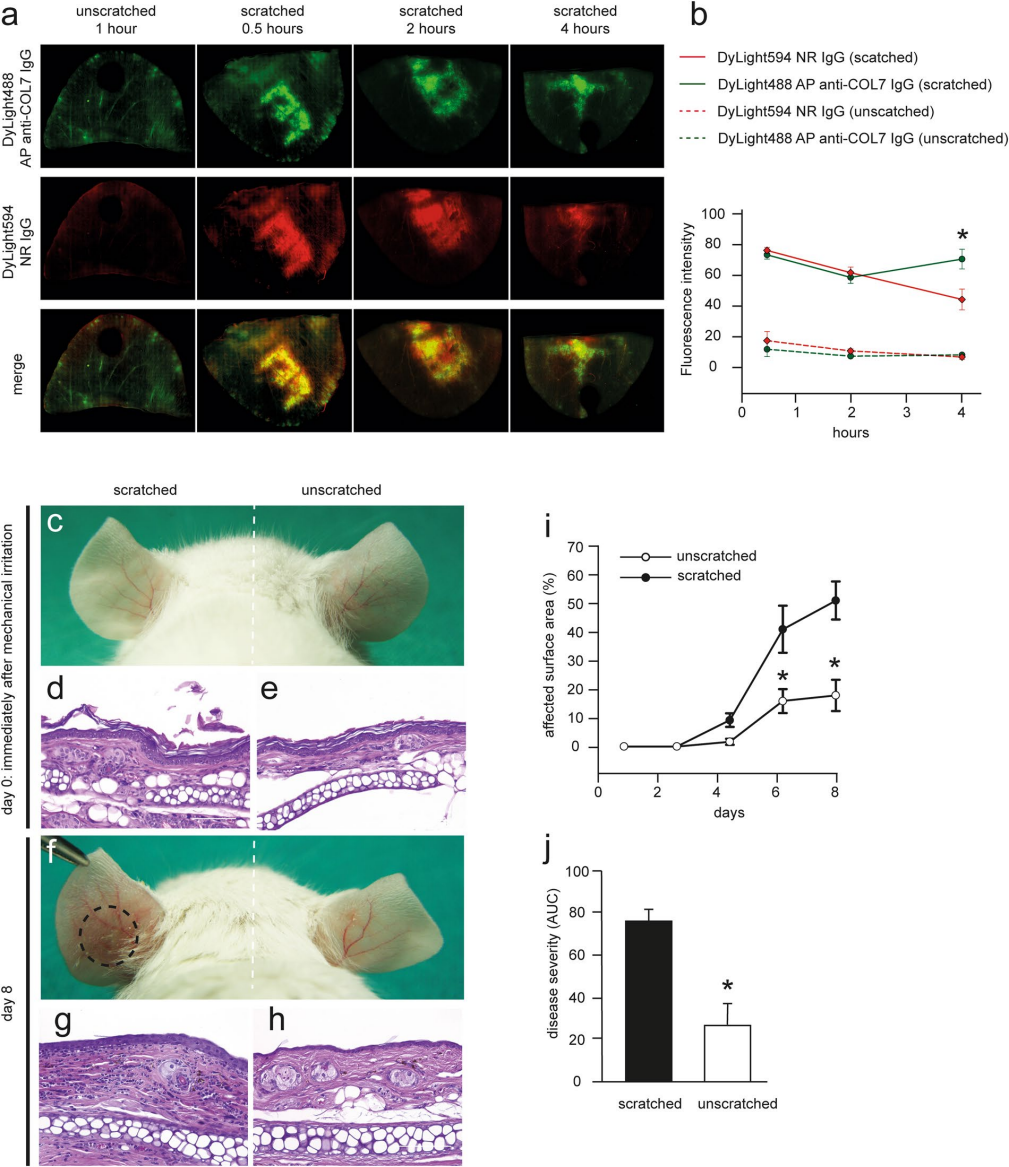
Mechanical irritation promotes anti-COL7 IgG deposition along the dermal-epidermal junction and enhances anti-COL7 transfer-induced skin blistering. (**a**) Images of ear specimens obtained on a specific time point after the i.v. injection of DyLight488-labeld anti-COL7 IgG or DyLight594-labeld normal rabbit IgG; scratched or non-scratched prior to IgG injection. (**b**) Fluorescent intensity determined by ImageJ software of the mouse ears (p < 0.05 comparison of t = 2 or 4 hours to t = 0.5 hours, RM ANOVA followed by Holm-Sidak for comparison to first time point). This finding indicates a significant (*p = 0.02, t-test) increase in the fluorescent intensity of anti-COL7 IgG. Data in a-b are based on 5 mice per group and time point. (**c**–**h**) Representative photos (**c**,**f**) and histological pictures (**d**,**e**,**g**,**h**) of mice s.c. with anti-COL7 IgG on day 0 and 8. (**i**) Percentage of affected surface area and (**j**) disease severity on day 8 are shown. The data are expressed as the mean ± SEM with an n = 3–5 mice per group. To compare the differences in the disease severity (AUC), independent samples Student’s t-tests were used. A p-value <0.05 was considered statistically significant.

Based on these findings, we hypothesized that local factors, such as mechanical irritation, may be the cause of the inhomogeneous deposition of anti-COL7 IgG. To challenge this assumption, ears of mice were gently scratched with tweezers prior to injection of antibodies. This procedure led to a rapid localization of both DyLight488 AP anti-COL7 IgG and control DyLight594 normal IgG in the skin, which was evidenced by strong fluorescent intensities. In contrast, un-manipulated contralateral ears exhibited substantially lower fluorescent intensities, even at later time points (Fig. 3a,b). Over a period of 4 hours, fluorescent intensity of the specific autoantibody remained constant, whereas fluorescent intensity of unspecific antibody gradually decreased in a linear fashion (Fig. 3a,b). To determine whether this increased IgG deposition in response to mechanical irritation is of functional relevance, experimental EBA was induced by repetitive injections of anti-COL7 IgG into BALB/c mice. In a subgroup of mice, macroscopically normal appearing ears (Fig. 3c) were obtained for histological analysis immediately after mechanical irritation. In the haemalaun eosin (H&E) stained sections from these specimens, no epidermal or dermal changes were evident (Fig. 3d,e). Interestingly, compared with the un-manipulated ears, the ears that had been mechanically irritated one time prior to anti-COL7 IgG injection developed more extensive blistering both clinically and histologically (Fig. 3f–j).

### Rapid dermal infiltration, but delayed co-localization with anti-COL7 IgG deposits, of LysM-eGFP^+^ cells

**Figure 4:**
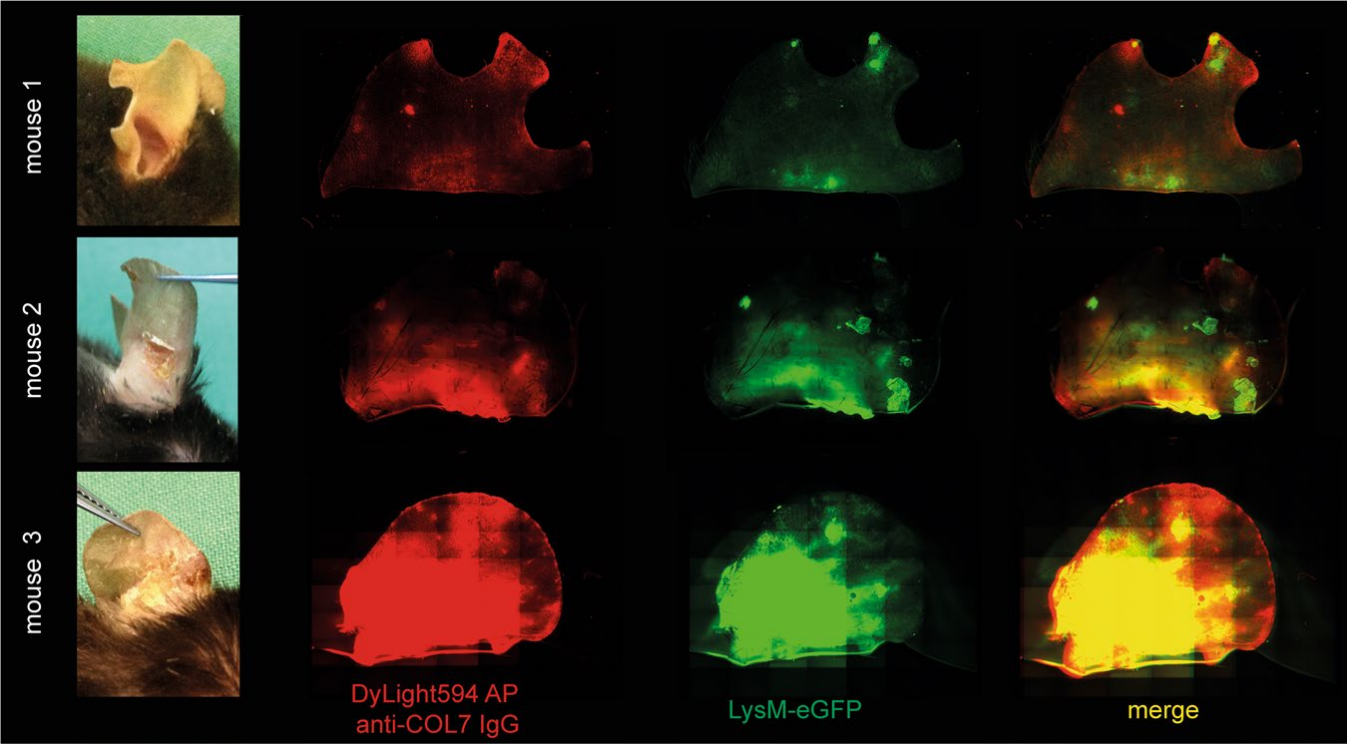
Co-localization of anti-COL7 IgG and myeloid cells in the skin of mice with experimental epidermolysis bullosa acquisita demonstrates presence of myeloid cells predominantly at the anti-COL7 IgG deposits. Experimental epidermolysis bullosa acquisita was induced in LysM-eGFP^+^ mice (green) via repetitive i.v. injections of DyLight594 affinity purified anti-COL7 IgG (red). The distribution of the antibody deposition and localization of the myeloid cell infiltrate were determined on day 9 in whole ears using fluorescent microscopy. In all mice, red fluorescence (which corresponds to antibody deposits) co-localized with the areas with clinical lesions. Furthermore, green fluorescence (which corresponds to myeloid cells) was predominantly present at the same sites. Three representative mice from a total of 9 are shown here. Of note as a standard procedure mice are notched at weaning. Therefore, the notches appear randomly due to this procedure (mouse 1: 2 notches, mouse 2: 1 notch, mouse 3: no notch).

In a subsequent set of experiments, we determined whether DyLight594 AP anti-COL7 IgG and LysM-eGFP^+^ cells (which corresponds to myeloid cells) co-localize in mice with antibody transfer-induced EBA. Thus, the images of whole ears were obtained 9 days after the initial anti-COL7 IgG injection (Fig. 4). One mouse exhibited only scant lesions on the investigated ear (Fig. 4, mouse #1), whereas the other animals presented with crusts and erosions located at the ears (Fig. 4, mouse #2 and #3). The infiltration with myeloid cells corresponded to the clinical presentation, and the green eGFP fluorescence (myeloid cells) corresponded to the red signal from the labeled AP anti-COL7 IgG (Fig. 4). To exclude the unspecific detection of fluorescence, the same experiment was conducted in C57Bl/6 mice (n = 3) injected with unlabeled anti-COL7 IgG. No fluorescence signal was identified in these mice.

**Figure 5:**
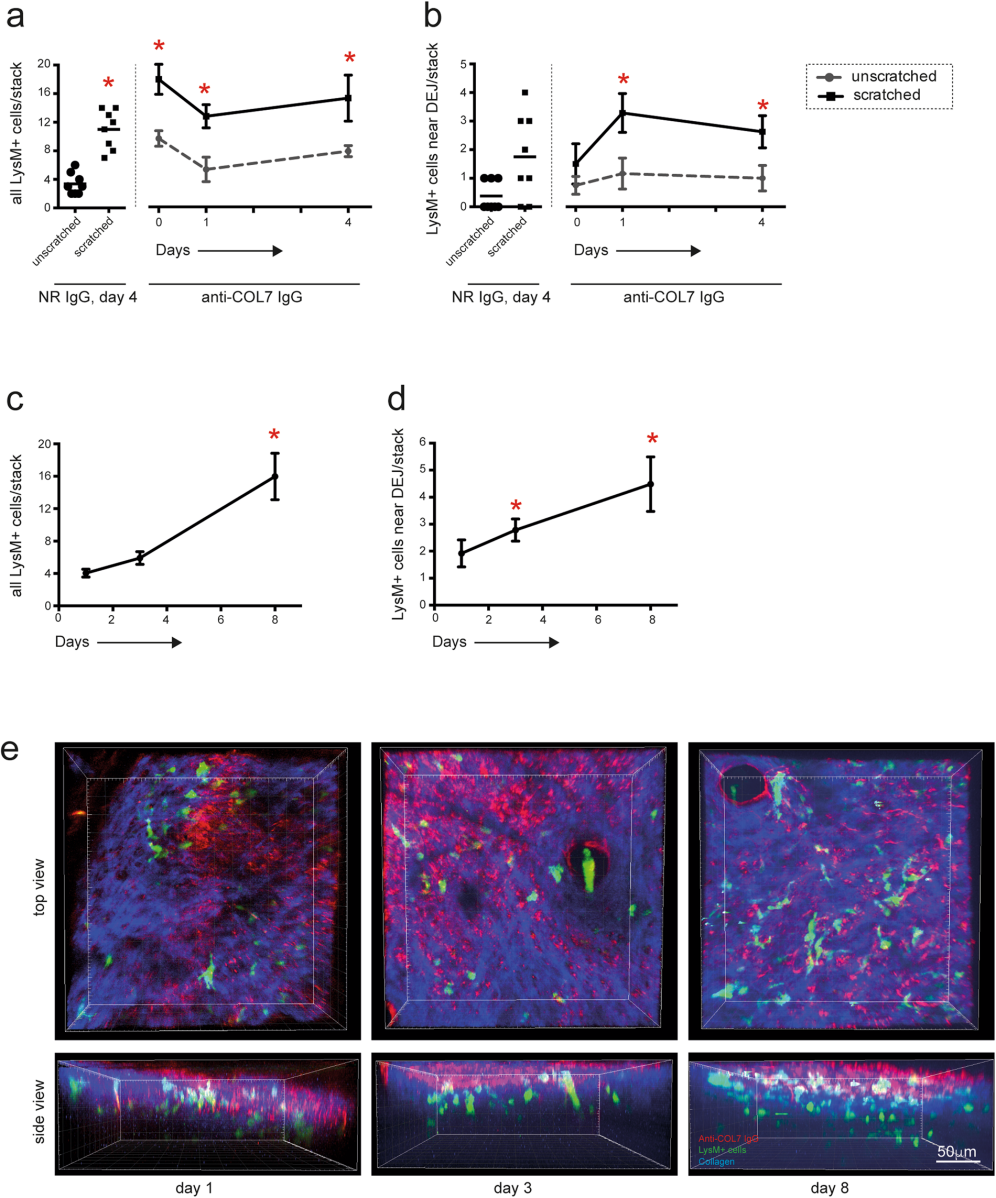
Rapid extravasation into the skin but delayed localization at the dermal-epidermal junction, of LysM-eGFP^+^ cells following anti-COL7 IgG injection. (**a**) Graph indicates the number of all LysM-eGFP^+^ cells identified in the dermal compartment. The bars correspond to the LysM-eGFP^+^ cells in the unscratched and scratched ears on day 4 after the initial normal rabbit IgG (NR IgG) i.v. injection and the lines correspond to the number of LysM-eGFP^+^ cells in the mice injected with anti-COL7 IgG on day 0, 1 and 4. The data are based on 6–8 evaluated stacks from at least 3 independently performed experiments [*p < 0.05, ANOVA on Ranks, followed by Dunn’s Method for comparison to reference (unscratched, normal rabbit IgG, day 4)]. (**b**) Here, only cells located at the dermal-epidermal junction were counted. (**c**) Total number of LysM-eGFP^+^ cells in the dermis and (**d**) number of LysM-eGFP^+^ cells located at the dermal-epidermal junction of mice i.v. injected with anti-COL7 IgG increases over the 8 day observation period. The data are based on experiments in 6 mice, and are presented as means and standard error for presentation purposes. (*p < 0.05, ANOVA on Ranks with Dunns’ Method, compared to day 1). (**e**) Representative stacks of the ears, generated from the images obtained by multiphoton microscopy.

We subsequently determined the kinetics of leukocyte recruitment into the skin in response to the injection of anti-COL7 IgG. DyLight594-labeled AP anti-COL7 IgG was injected into mice immediately prior to multiphoton imaging. The behavior of the LysM-eGFP^+^ cells in the individual mice was visualized directly after 2 hours as well as 1, 3, 4 or 8 days after the initial IgG injection (the mice investigated for 4 days after the initial IgG injection received a second IgG dose at day 2) (Fig. 5). Within the first two hours after the IgG injection, the total number of LysM-eGFP^+^ cells located within the dermal compartment moderately increased in the ears of the mice that had been mechanically irritated one time prior to the initial IgG injection. This increase in the LysM-eGFP^+^ cell numbers in the mechanically irritated ears was maintained throughout the observation period (Fig. 5a). In contrast, in the un-manipulated ears, no increase in the LysM-eGFP^+^ cell numbers was identified at any time point (Fig. 5a). Considering LysM-eGFP^+^ cells located in the vicinity of the DEJ, i.e., at the site of IgG deposition, a significant increase was identified with a delay of 24 hours after the initial influx of LysM-eGFP^+^ cells in the animals injected with anti-COL7 IgG, as well as the ears that had been mechanically irritated (Fig. 5b, supplementary Video V1). This finding of a delayed binding of LysM-eGFP^+^ cells to the immune-complexes located at the DEJ is also supported by H&E (or Gr-1) stained ear specimens obtained from mice with antibody transfer-induced EBA, in which the dermal infiltration with Gr-1 myeloid cells is predominately located below, and not at, the DEJ (supplementary Fig. S2). The injection of normal rabbit IgG led to an increase in the total LysM-eGFP^+^ cell influx in the mechanically irritated ears; in contrast, no increased localization of cells to the DEJ was identified (Fig. 5a,b, supplementary Videos V2-5).

To obtain longitudinal insights into the migratory behavior of LysM-eGFP^+^ cells, as well as for the investigation of later time points, LysM-eGFP^+^ mice were injected with DyLight594-labeled AP anti-COL7 IgG, and single mice were investigated 1, 3 and 8 days after the initial IgG injection. A further increase in LysM-eGFP^+^ cells into the dermis was identified 8 days after the initial IgG injection (Fig. 5c,e). Consistent with our previous findings, the influx of LysM-eGFP^+^ cells increased over time, and a further increase in LysM-eGFP^+^ cells at the DEJ was identified on day 8 (Fig. 5d,e). Collectively, the neutrophil influx postdates IgG deposition by at least 24 hours (Fig. 5a). The location of LysM-eGFP^+^ cells to IgG deposits located at the DEJ follows the extravasation of these cells into the skin. Furthermore, the location of LysM-eGFP^+^ cells to the site of immune complex deposition is not permanent, but rather short-lived (supplementary Videos V2-5).

### Antibody transfer-induced EBA leads to a late-onset increased vascular leakage

**Figure 6:**
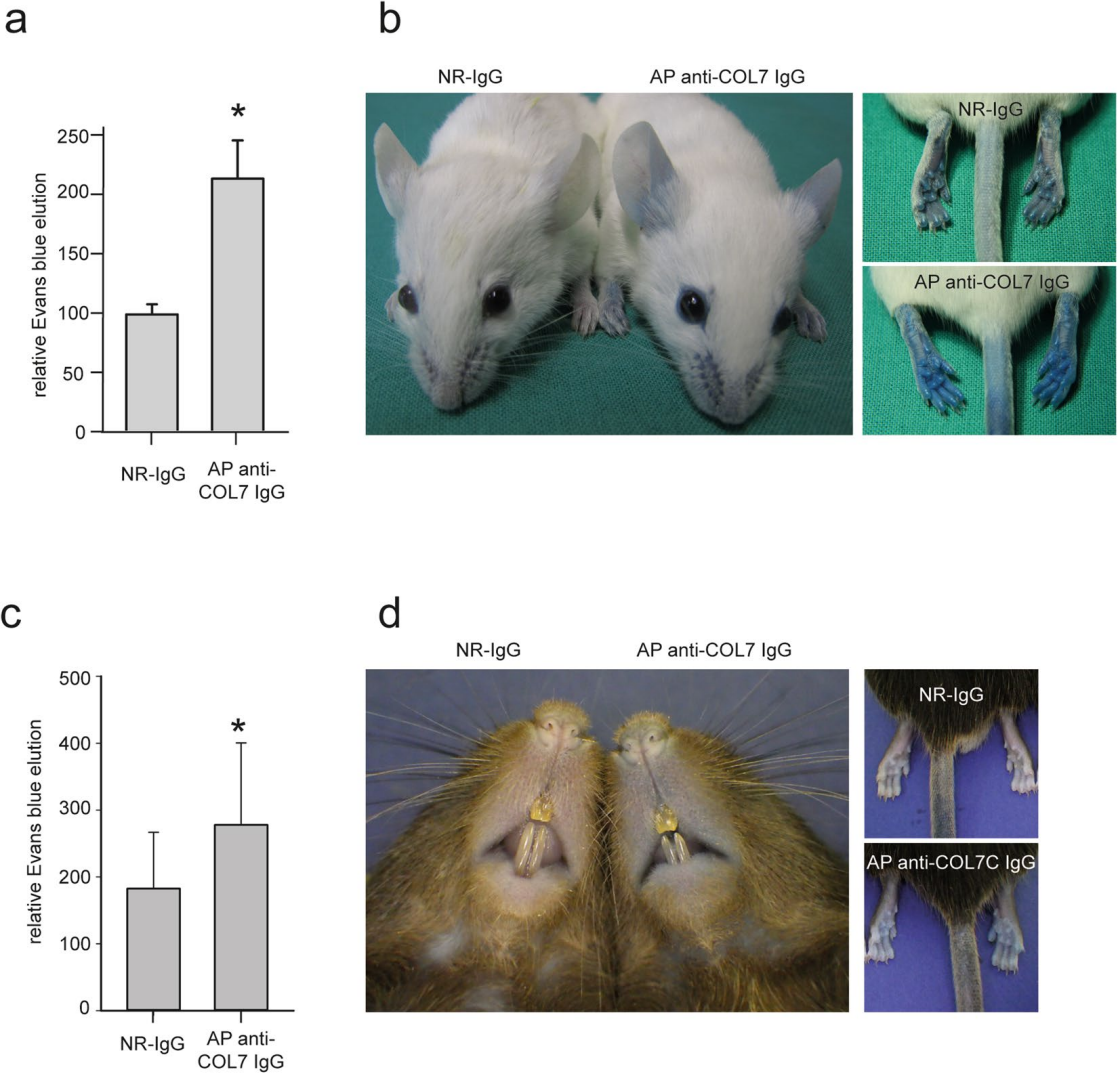
Late-onset increased vascular permeability in experimental epidermolysis bullosa acquisita. (**a**,**c**) Mean (SEM) Evans blue elution (in relation to weight) in BALB/c (**a**) and C57BL/6J (**c**) mice injected s.c. with normal rabbit IgG (NR-IgG) or affinity-purified anti-COL7 IgG 4 (AP anti-COL7 IgG) days after the initial IgG injection. The induction of experimental experimental epidermolysis bullosa acquisita led to increased vascular permeability, which was evidenced by an increase in eluted Evans blue (*p < 0.05, t-test, n = 4–5 mice/group). (**b**,**d**) Representative clinical images of BALB/c (**b**) and C57BL/6J (**d**) mice injected with NR-IgG or AP anti-COL7 IgG and Evans blue.

Swelling, which is caused by increased vascular permeability and leukocyte infiltration, is one hallmark of inflammation, and increased vascular permeability may represent an early event in the pathogenesis of specific inflammatory diseases, such as arthritis^38^. We therefore investigated whether and when an increase in the vascular permeability may occur in experimental EBA. Of note, without prior mechanical irritation, an increased vascular permeability, visualized by Evans blue, was only identified 4 days after the initial anti-COL7 IgG injections in BALB/c mice (Fig. 6a,b); at the previous time points, no enhanced dye extravasation was seen in the anti-COL7 IgG compared with the normal rabbit IgG injected mice. Similar findings were obtained in C57BL/6J mice (Fig. 6c,d)

## Discussion

Overall, we define checkpoints for autoantibody-induced tissue injury exemplified in EBA: (i) autoantibody egress to the target tissue, which is influenced by mechanical trigger factors, (ii) neutrophil recruitment into the vicinity of autoantibody deposits within hours of immune-complex formation, and (iii) delayed massive neutrophil localization to these deposits and subsequent autoantibody-induced tissue damage.

The binding of autoantibodies to COL7 is without doubt the initiating event (and first checkpoint) that leads to inflammation and blistering in EBA^25,39^. Deposits of IgG and/or IgA in the skin are identified in 95% of patients at the time of diagnosis^40–42^. Furthermore, a detailed morphological analysis in antibody transfer-induced EBA indicated anti-COL7 binding to the DEJ as early as 24 hours after subcutaneous injection^43^. Nevertheless, detailed insights into the dynamics of anti-COL7 binding have not been reported. The current study demonstrates that in several areas of skin, IgG, independent of the specificity, rapidly leaves the circulation within several minutes and is present in the skin of healthy mice. COL7-specific antibodies are subsequently retained in the skin in contrast to non-specific antibodies (Fig. 3b). This observation is consistent with data obtained in anti-GPI transfer-induced arthritis, in which anti-GPI IgG specifically located at the site of disease manifestation (i.e., the joints) within minutes, despite the ubiquitous GPI expression and systemic circulation of anti-GPI IgG. Interestingly, in this study, unspecific control antibodies were not identified in the joints^29^. This finding may be explained by the different methods used and a higher sensitivity of multiphoton microscopy used in the current study compared with positron emission tomography used in the arthritis study. Thus, we envision that IgG deposits in the skin by diffusion, as previously reported^44,45^. The antibodies are only retained in the skin if they bind to specific receptors, i.e., the high affinity IgE receptor expressed by cutaneous mast cells or, such as in our case, if they specifically bind to a target (auto)-antigen expressed in the skin. Moreover, in contrast with antibody transfer-induced arthritis^38^, the localization of anti-COL7 IgG into the skin is most likely not modulated by a rapid induction of histamine- and serotonin-triggered macromolecular vasopermeability because an enhanced Evans blue localization was only identified 4 days after the initial IgG injection. Nevertheless, the observation of an increased anti-COL7 IgG deposition following mechanical irritation suggests yet-to-be-defined molecular mechanisms that control the permeability of IgG into the skin. This finding may be explained by the close interaction of nociceptive sensory neurons in the skin and their essential roles in driving specific inflammatory processes, such as experimental psoriasis^46^. Regarding the distribution of the anti-COL7 IgG, we assumed a homogenous and linear binding along the DEJ. In contrast to our expectations, a patchy binding was identified. This (at least initially) inhomogeneous distribution of the anti-COL7 IgG may reflect that different mechanical irritations occur at various sites.

The second checkpoint in antibody-induced tissue inflammation and tissue injury is the extravasation of myeloid effector cells into the tissues^35,47^. In EBA, the binding of the autoantibodies to their respective target antigen induces a pro-inflammatory microenvironment that consists of cytokines and complement, which facilitate a CD18/ICAM-depended extravasation of myeloid effector cells^28,35,48–50^. The source of these mediators must be further defined. One potential candidate to produce (at least some of) the mediators that lead to myeloid cell extravasation are keratinocytes, which have been shown to release IL-6 and IL-8 following incubation with autoantibodies that target another structural protein of the skin, namely, type XVII collagen^51^. The delayed (in relation to the anti-COL7 IgG binding) extravasation into the dermis in the current study was expected. The identification of the source of these mediators and their respective control of myeloid cell extravasation will hopefully enable the identification of novel and more specific therapeutic targets for the treatment of EBA and diseases with a similar pathogenesis.

Many studies have been published regarding the adhesion molecules involved in the interactions of leukocytes with endothelial cells. In contrast, surprisingly little information is available regarding the epithelial localization of leukocytes^52^. Similar considerations apply for the extravasation of neutrophils into the skin and their subsequent movements in the skin^53^. In experimental EBA, myeloid effector cells bind to the immune complexes in a specific Fc gamma receptor-dependent manner in both mice and humans^54,55^. This binding leads to a yet-to-be-defined signaling cascade within the myeloid cells that involves PI3K beta and delta, as well as, AKT, p38 MAPK, AKT, Src family kinases, SYK, and RORα^56–63^. Ultimately, myeloid cells release proteases^64^ and ROS^35^, which, in turn, cause subepidermal blistering and may further enhance the influx of myeloid cells into the skin^65^. Based on these considerations, we thus expected prolonged contacts of LysM-eGFP^+^ cells with the antibody deposits. Of note, despite their location close to the DEJ, only a few of these cells were identified at the site of antibody deposits. Furthermore, if this interaction occurred, it was short-lived. This prompted us to re-investigate conventionally stained H&E skin specimens from mice with antibody transfer-induced EBA. Consistent with the current findings, the majority of the dermal infiltrate was located not in direct contact with the DEJ. Therefore, these rather few and short-lived interactions appear to suffice to induce subepidermal blistering. In the future, the use of fluorescence probes to specifically detect ROS^66^ may facilitate the further unraveling final checkpoint of autoantibody-induced tissue damage.

The simultaneous observation of autoantibodies and leukocytes in an organ-specific autoimmune disease provided novel and unexpected insights into the pathogenesis of autoantibody-induced inflammation and tissue injury, which were exemplified by EBA. Our data also enabled the identification of precise checkpoints that lead to inflammation and blistering in EBA. Unraveling the molecular basis of each checkpoint will aid in the definition and identification of (hopefully more selective) therapeutic targets.

## Methods

### Animal studies

C57Bl/6J and BALB/c mice were obtained from The Jackson Laboratories (Bar Harbor, ME, USA). LysM-eGFP mice were kindly provided by Dr. Graf^36^. The animals were fed acidified drinking water and standard chow ad libitum and were maintained on a 12 h light-dark cycle at the animal facility of the University of Lübeck. The experimental animal protocols were approved by local authorities of the Animal Care and Use Committee of the Federal Ministry of Food and Agriculture of Schleswig-Holstein (Kiel, Germany; 2-1/11, 72-7/10) and were performed by certified personnel. We confirm that all methods were performed in accordance with the relevant guidelines and regulations.

### Generation of affinity-purified, fluorescently labeled anti-COL7 IgG

Rabbit immunization was performed following previous protocols with minor modifications^37,67^. Briefly, two New Zealand White rabbits (SA6306 and SA6307) were subcutaneously immunized with 250 μg of murine GST-mCOL7C^37^ suspended in Freund’s complete adjuvant. The animals were boosted three times (at 13-day intervals) with the same protein preparation in incomplete Freund’s adjuvant. The immune sera were characterized via immunofluorescence (IF) microscopy using cryosections of murine skin. The total IgG from the immune and normal rabbit sera was purified via affinity chromatography using protein G affinity as previously reported^37^. COL7 specific IgG was subsequently isolated from purified total IgG using Affi-Gel (Bio-Rad, Munich, Germany). Briefly, to prepare the specific column, 10 mg of recombinant COL7 protein in 0.1 M MOPS buffer, pH 7.5, that contained 80 mM CaCl_2_ was coupled with Affi-Gel 10 according to the manufacturer’s instructions. Three hundred mg total IgG was passed through the specific column 5 times under a gravity condition, and the column was subsequently washed with 50 ml of PBS, 50 ml of PBS that contained 850 mM NaCl and 0.1% Triton X-100 (Merck, Darmstadt, Germany), followed by an additional 50 ml of PBS. COL7 specific IgG was subsequently eluted with 0.1 M glycine buffer, pH 2.8, and immediately adjusted to pH 7.2 with 1 M Tris. Specific IgG was concentrated, and the buffer was changed to PBS via Centricons (Millipore, Billerica, USA). Following passage through a 0.2-µm filter (Sartorius Stedim Biotech, Gottingen, Germany), the concentration was adjusted to 2.0 mg/ml for affinity purified specific anti-COL7 IgG (AP anit-COL7 IgG). For labeling with a fluorescent dye, AP-IgG or normal rabbit IgG were conjugated with DyLight488 or DyLight594 (Thermo Scientific, Waltham, USA) according to the manufacturer’s instructions for these antibody labeling kits.

### Determination of *ex vivo* blister-inducing capacity of anti-COL7 IgG

The split-inducing capacities of total IgG, AP anti-COL7 IgG, DyLight488 AP anti-COL7 IgG, and DyLight594 AP anti-COL7 IgG were determined using an *in vitro* assay as reported by^68^. Briefly, cryosections prepared from mouse back skin (were placed in the center of a Superfrost Plus microscope slide (Menzel-Glaser, Braunschweig, Germany). Skin sections were washed with PBS for 5 minutes to remove embedding medium, then incubated with the above indicated antibody preparations for 90 minutes at room temperature. After washing the sections with PBS, mouse PMNs were added to the sections. Incubation of PMNs with skin sections was performed in a humidified air incubator containing 5% CO_2_ for 3 hours at 37 °C. Subsequently, sections were washed in PBS, fixed in formalin, and stained with hematoxylin and eosin. Finally, skin dermal-epidermal separation was evaluated by an investigator unaware of the section’s treatments. To compare the pathogenicity of total IgG and AP anti-COL7 IgG, the concentrations were adjusted based on the titer levels, which were determined via indirect IF microscopy.

### Anti-COL7 IgG transfer-induced experimental epidermolysis bullosa acquisita in mice

For the *in vivo* pathogenicity test, normal IgG, total anti-COL7 IgG (7.5 mg/injection), AP anti-COL7 (75 or 150 µg/injection), DyLight488 AP anti-COL7 IgG, and DyLight594 AP anti-COL7 IgG (75 or 150 µg/injection) were subcutaneously injected into adult C57Bl/6J mice every second day for a total of 6 injections. The mice were assessed for their general condition and evidence of cutaneous lesions (i.e., erythema, blisters, erosions, alopecia and crusts) every fourth day and were subsequently assessed until 12 days after the initial injection. In some experiments, one ear was gently mechanically irritated with forceps at day 0 prior to the IgG injection. 200 µg of AP anti-COL7 IgG was subsequently injected i.p. at days 0 and 2, and the affected areas (%) of both ears were scored every second day until day 8.

### Immunofluorescence microscopy and multiphoton imaging

To analyze the distribution and the kinetics of pathogenic IgG, organ samples from the entire body (tongue, esophagus, stomach, small intestine, colon, trachea, lung, brain, heart, spleen, liver, kidney, uterus, and muscle) were obtained at 1, 4, 8–12, 20–24, and 44–48 h following injection into the tail vein of 250 µg of DyLight488 AP anti-COL7 IgG, DyLight488 labeled normal rabbit IgG or DyLight488 AP anti-COL7 IgG together with DyLight594 labeled normal rabbit IgG. 6 µm cryosections were prepared from all organs. The sections were subsequently washed with PBS (some sections were also stained with DAPI), and tissue-bound IgG was detected via fluorescence microscopy. Whole ears from the LysM-eGFP mice, which were previously used for multiphoton imaging, were obtained after observation on day 9. The whole ears were examined under a fluorescent microscope, Biozero BZ-9000E (Keyence, Osaka, Japan), and images of the whole ears were obtained for the signals of DyLight594 AP anti-COL7 IgG (red) and eGFP^+^ cells (green, from LysM-eGFP mice). Using the analysis software BZ-II Analyzer (Keyence, Osaka, Japan), an overlay of the signals was created.

LysM-eGFP mice^36^ received two i.v. injections (days 0 and 2) of 250 µg (2 mg/ml) of DyLight594 labeled AP rabbit anti-COL7 IgG. These events led to inflammation and blisters in the antibody transfer-induced EBA. The migration behaviors of the Lys-eGFP^+^ neutrophils and monocytes were further investigated, i.e., the LysM-eGFP mice were anesthetized via injection for long-term experiments (mixture of 50 µl Fentanyl, 400 µl Midazolam, 200 µl Dormitor and 3.5 ml Ringer solution). Hair removal cream (GlaxoSmithKline, Bühl, Germany) was subsequently applied on the ears to remove the hair shafts. Imaging was performed using a TriM Scope II multiphoton microscope (LaVision BioTec GmbH, Bielefeld, Germany) equipped with a XLUMPLFL 20× W/0.95 objective (Olympus, Hamburg, Germany)^69^. The excitation wavelength for the visualization of anti-COL7 IgG was 740 nm. EGFP and the second harmonic generation signal for collagen were excited with 900 nm. The laser intensity was optimized to reliably visualize only bright eGFP cells to reduce possible laser damage from excitation of melanin. Since the eGFP signal in monocytes is considerably weaker than in neutrophils, (see supplementary Fig. S3) this setting preferentially visualized neutrophils but detection of monocytes cannot be excluded. The emitted light was detected by three wavelength-separated PMTs (435–495, 495–560 and >560 nm). Visualization of the migratory behavior of eGFP^+^ cells in the ear skin was performed between days 0 and 8 after the initial anti-COL7 IgG injection for the induction of experimental EBA. Seven different Z-stacks were acquired at each time point of the experiment for each mouse. Each stack had a size of 250 × 250 × 90 µm. Image processing was conducted using Imaris Software (Bitplane, Zürich, Switzerland), and the total number of extravasated eGFP^+^ cells was counted per eye in the whole Z-stack or the vicinity of 16 µm below the DEJ. Cells that were identified in the dermis were considered extravasated.

In another experiment, BALB/c mice were anesthetized as previously described, and hair removal cream (GlaxoSmithKline, Bühl, Germany) was subsequently applied to the ears to remove the hair shafts. The surface of one ear was gently scratched using forceps. Multiphoton imaging was performed using a TriM Scope II multiphoton microscope (LaVision BioTec GmbH, Bielefeld, Germany). First, the ears were assessed prior to IgG injection. 250 µg (2 mg/ml) of DyLight488 AP anti-COL7 IgG or normal rabbit IgG were subsequently injected into the tail vein, and the ears were immediately assessed with the microscope. In some experiments, 125 µg (2 mg/ml) of DyLight488 AP anti-COL7 IgG and 125 µg (2 mg/ml) of DyLight594 normal rabbit IgG were injected.

### Immunohistological studies

Skin samples were fixed in 4% buffered formalin and embedded in paraffin. Four µm thick sections of the ears were cut and stained for H&E as previously described^70^. Cryostat sections of the ear skin were stained for granulocytes. Briefly, a monoclonal antibody against Gr-1 (Ly6C/G, RBC-8C5, eBioscience) was used as the primary antibody, and an alkaline phosphatase-conjugated goat anti-rat IgG (Roth, Karlsruhe, Germany) comprised the secondary antibody. A Biozero BZ-9000E from Keyence (Keyence, Osaka, Japan) was used for light microscopy.

### Analysis of vascular leakage

To investigate the change of vascular permeability in diseased mice, 100 µl of 1% Evans blue (Sigma, St. Louis, USA) in PBS were sterilized by passage through a 0.2 µm filter (Sartorius Stedim Biotech, Göttingen, Germany) and were subsequently injected into the tail vein of the mouse. At the same time, 250 µg of AP anti-COL7 IgG were injected i.p. BALB/c and C57BL/6J mice were assessed every day until day 5. On day 5, both ears and both hind legs were harvested and dried at room temperature for 3 days. The tissues were subsequently weighed and incubated with 100 µl of formamide (Sigma-Aldrich, Munich, Germany) at 70 °C overnight to elute Evans blue. The absorbance was measured using NanoDrop spectrophotometer at a 620 nm wavelength, and the concentration of Evans blue was calculated from a standard curve of Evans blue in formamide. To investigate the impact of scratching irritation for vascular permeability, DyLight488 AP anti-COL7 IgG (100 µg) and DyLight594 normal rabbit IgG (100 µg) were injected into the tail vein with and without mechanical irritation. After 0.5 h and up to 2 days, the ears were harvested and assessed using fluorescence microscopy (Keyence, Osaka, Japan).

### Statistical analysis

The data are expressed as the mean ± SEM. To compare the concentrations of Evans blue and the differences in the disease severity (AUC), independent samples Student’s t-tests were used. For the analyses of eGFP^+^ cells, one-way ANOVA and the appropriate post hoc tests were used. A p-value <0.05 was considered statistically significant.

### Approval

All experimental protocols were approved by a named licensing committee. Please identify the approving body in the methods section.

## Supplementary information

Supplementary video 1.

Supplementary video 2.

Supplementary video 3.

Supplementary video 4.

Supplementary video 5.

Supplementary figures.

## Data Availability

Authors make all data underlying the findings described in their manuscript fully available without restriction.
