# Supplementary material for: Visualization of autoantibodies and neutrophils in vivo identifies novel checkpoints in autoantibody-induced tissue injury
Source: Sci Rep. 2020 Mar 11;10:4509. doi: 10.1038/s41598-020-60233-w (PMC7066238; doi:10.1038/s41598-020-60233-w)
Supplement: Supplementary file 6 — Supplementary figures. [file 41598_2020_60233_MOESM6_ESM.doc]

**SUPPLEMENTARY INFORMATION**

**Visualization of autoantibodies and neutrophils in vivo identifies novel checkpoints in autoantibody-induced tissue injury**

**Jennifer E. Hundt**^1,+^, **Hiroaki Iwata**^2,+,§^**, Mario Pieper**^3,+,*^**, Rebecca Pfündl**^1^, **Katja Bieber**^1^**, Detlef Zillikens**^1,2^**, Peter König**^3,+^**, and Ralf J. Ludwig**^1,2,+^

^1^Institute of Experimental Dermatology, University of Lübeck, Germany

^2^Department of Dermatology, University of Lübeck, Germany

^3^Insitute of Anatomy, University of Lübeck, Germany and Airway Research Center North (ARCN), Member of the German Center for Lung Research (DZL)

^+^These authors contributed equally to this work (alphabetical order)

^§^Current address: Department of Dermatology, Hokkaido University Graduate School of Medicine, Sapporo, Japan

^*^Corresponding author


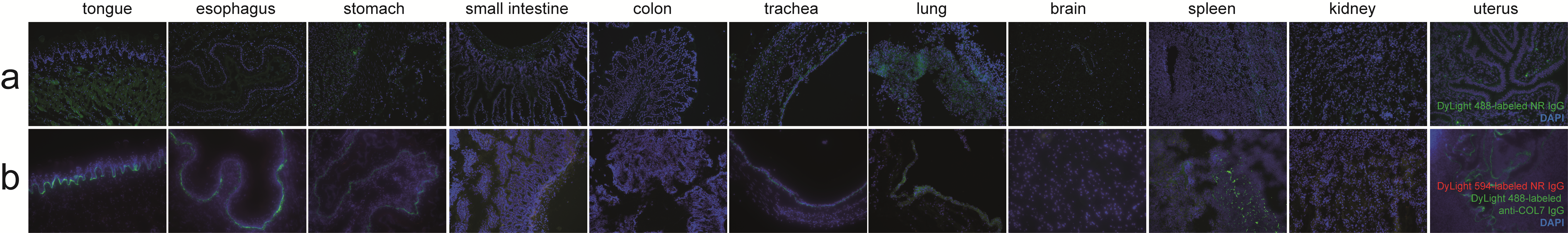


**Supplementary Figure S1. *In vivo* binding patterns of anti-COL7 IgG**

(a) To control for unspecific binding, normal rabbit IgG was labelled with DyLight488 and injected i.v. into the mice. Ten hours after the IgG injection, organs were obtained and evaluated for fluorescence. No specific IgG was detected in a total of 3 independent experiments. (b) To control for the labelling, Dylight594-labeled normal rabbit IgG was co-injected with DyLight 488-labeled anti COL7 IgG. Again, similar staining patterns were observed in 3 animals.


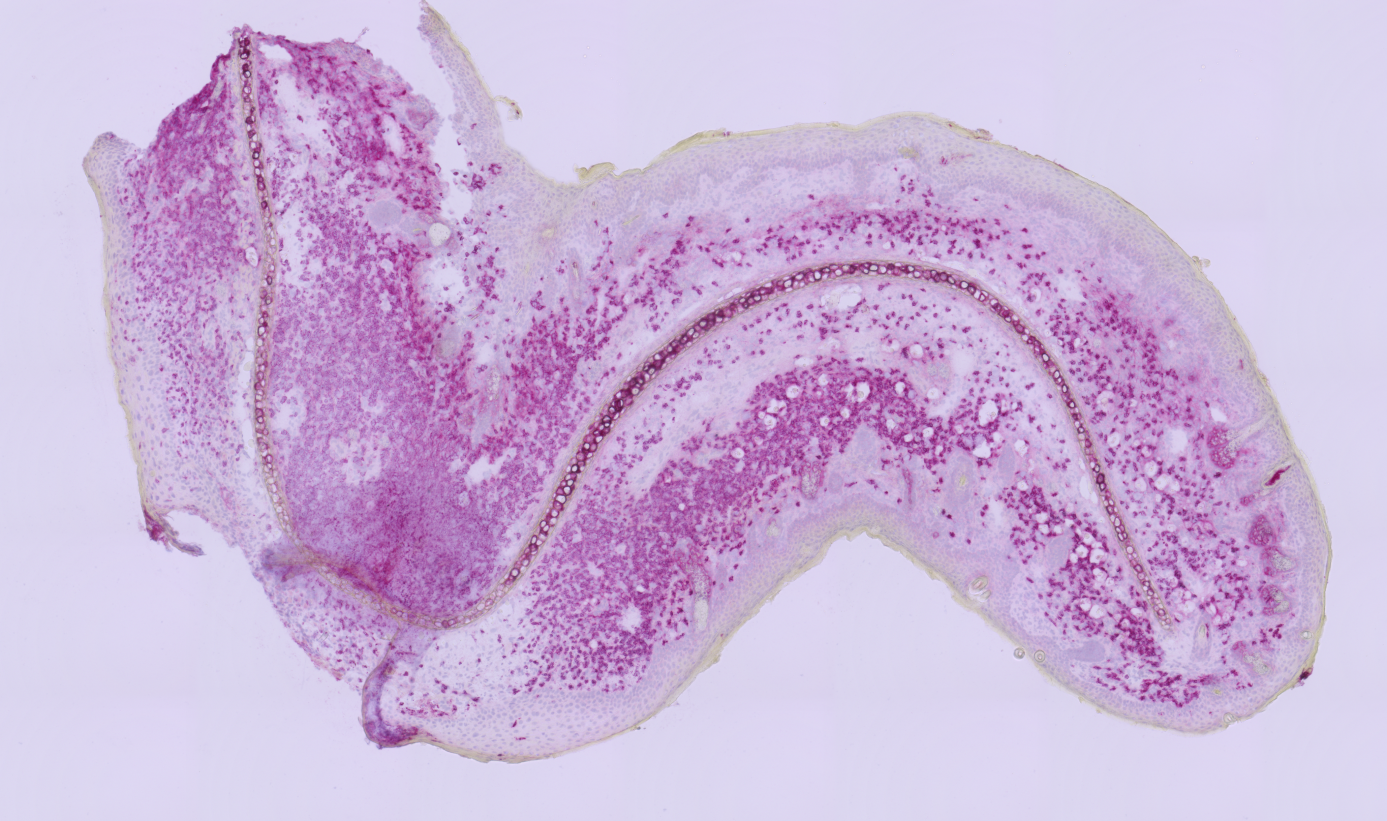


**Supplementary Figure S2. Dermal infiltration localizes in lower dermis in mice with antibody transfer-induced epidermolysis bullosa acquisita**

Gr-1 stained specimen from a mouse with antibody transfer-induced epidermolysis bullosa acquisita (s.c. injected anti-COL7 IgG), obtained at day 12 of the experiment. The dermal infiltrate is predominately located in the lower dermis; in a limited number of cases, (Gr-1+) leukocytes are located at the dermal-epidermal junction.


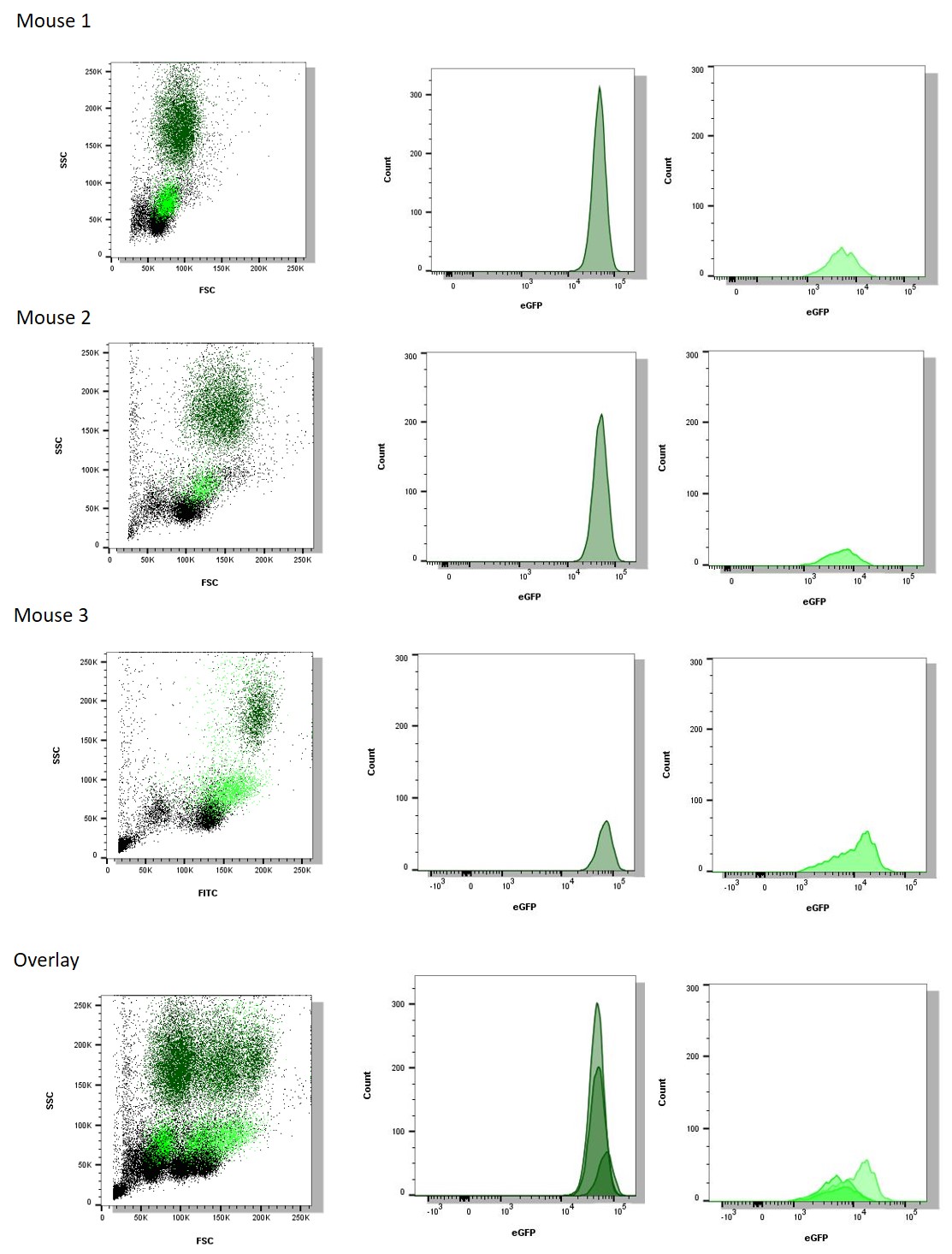


**Supplementary Figure S3. Differentiation between neutrophils and monocytes in LysM-eGFP mice using fluorescence-activated cell scanning analyses**

Fluorescence-activated cell scanning (FACS) blood analyses of LysM-eGFP mice (n= 3) are shown here for each mouse 1-3 and as an overlay for all 3 mice. Neutrophils (dark green) are well distinguishable in the forward and sideward scatter presentation on the left hand side due to the maximized size and the higher granularity compared to monocytes (light green). Neutrophils also differ due to their higher fluorescent signal on the (x-axis, middle graphs) compared to monocytes (x-axis, right hand side graphs). In mouse 1 and 2 the neutrophil cell count (y-axis, middle graphs) is higher compared to the number of monocytes (y-axis, right hand side graphs); in mouse 3 the amount of neutrophils and monocytes is similar.

**Description of video files**

**Supplementary video 1**

A representative 3D stack from day 8 after IgG i.v. injection (250 µm x 250 µmx 90 µm of size) of a mouse ear generated from the images obtained using multiphoton microscopy is shown here rotating. LysM-eGFP+ cells (green) are located in the vicinity of the dermal-epidermal, i.e., at the site of IgG (red) deposition, and the second harmonic generation signal of collagen (blue) in the dermis.

**Supplementary video 2**

A representative video of a 3D stack of a mouse ear from day 4 after unspecific IgG i.v. injection generated by multiphoton microscopy is shown here. A low amount (ear unscratched) of migrating LysM-eGFP+ cells (green) is visible within the dermis (blue due to second harmonic generation signal of collagen). Unspecific IgG (red) does not bind to the dermal-epidermal junction and is therefore not visible.

**Supplementary video 3**

A representative video of a 3D stack of a mouse ear from day 4 after unspecific IgG i.v. injection generated by multiphoton microscopy is shown here. A higher amount (ear scratched) of migrating LysM-eGFP+ cells (green) is visible within the dermis (blue due to second harmonic generation signal of collagen). Unspecific IgG (red) does bind to the dermal-epidermal junction and is therefore not visible.

**Supplementary video 4**

A representative video of a 3D stack of a mouse ear from day 4 after anti-COL7 IgG i.v. injection generated by multiphoton microscopy is shown here. A low amount (ear unscratched) of migrating LysM-eGFP+ cells (green) is visible within the dermis (blue due to second harmonic generation signal of collagen). Specific IgG anti-COL7 (red) binds to the dermal-epidermal junction and is visible.

**Supplementary video 5**

A representative video of a 3D stack of a mouse ear from day 4 after anti-COL7 IgG i.v. injection generated by multiphoton microscopy is shown here. A hig amount (ear scratched) of migrating LysM-eGFP+ cells (green) is visible within the dermis (blue due to second harmonic generation signal of collagen). Specific IgG anti-COL7 (red) binds to the dermal-epidermal junction and is visible.
